# Direct whole-genome sequencing enables strain typing of unculturable Neisseria meningitidis from oropharyngeal carriage specimens

**DOI:** 10.1099/mgen.0.001464

**Published:** 2025-08-04

**Authors:** Rosa C. Coldbeck-Shackley, Andrew Lawrence, Mark McMillan, Caitlin A. Selway, Lito Papanicolas, Mark Turra, Helen Marshall, Lex E. X. Leong

**Affiliations:** 1 Microbiology and Infectious Diseases, SA Pathology, Adelaide 5000, Australia; 2 Vaccinology and Immunology Research Trials Unit, Women’s and Children’s Health Network, Adelaide 5000, Australia; 3 Robinson Research Institute and Adelaide Medical School, The University of Adelaide, Adelaide 5000, Australia; 4 UniSA Clinical and Health Sciences, University of South Australia, Adelaide 5000, Australia

**Keywords:** *Neisseria meningitidis*, metagenomics, typing, whole-genome sequencing

## Abstract

Oropharyngeal carriage of *Neisseria meningitidis* (*N.m*.) is a prerequisite for invasive meningococcal disease. As such, genomic surveillance of disease-causing carriage strains can inform targeted public health responses. However, whole-genome sequencing (WGS) from isolates is often precluded due to the high rates of culture failure for *N.m*. samples collected in carriage studies. This study outlines an alternative method to sequence *N.m*. directly from oropharyngeal specimens that enables high-resolution molecular fine typing.

We performed direct probe-capture enrichment WGS (dWGS) of *N.m*. on oropharyngeal specimens from the ‘B part of it’ South Australian and ‘B part of it NT’ Northern Territory meningococcal carriage studies (NCT03089086 and NCT04398849). Sequences were analysed using currently available bioinformatic tools, including the characterization of genogroup, multi-locus sequence typing (MLST), *Bexsero* Antigen Sequence Typing (BAST), *porA* and *fetA* type.

Sensitivity of dWGS typing compared to WGS for genogroup, MLST, *porA*, *fetA* and BAST schemes was 88.89%, 72.22%, 100%, 94.44% and 88.24%, respectively. Genogroup and *porA* type were more reliably characterized in unculturable samples compared to the other typing schemes assessed. Factors that influenced accurate fine typing included the amount and proportion of *N.m*. sequences, and the proportion of other *Neisseria* species in enriched sequencing libraries. An alternative phylogenetic method (phylotyping) correctly predicted the clonal complex for 93.46% of the samples assessed. These results demonstrate that dWGS enables high-resolution molecular fine typing and can be applied to unculturable samples in *N.m*. carriage studies.

**Impact Statement**

Although MenB vaccines effectively protect against invasive meningococcal disease caused by serogroup B *Neisseria meningitidis* (*N.m.*), growing evidence suggests that they do not prevent carriage of serogroup B meningococci and are, therefore, unlikely to prevent their transmission [6,47]. This is distinct from vaccines targeting serogroup ACYW meningococci that also prevent their carriage. Ongoing surveillance of *N.m*. carriage could help to inform targeted MenB vaccination in high-risk groups. However, *N.m*. carriage studies are often hampered by high rates of culture failure for this fastidious organism, precluding whole-genome sequencing typing in a significant proportion of samples. Here, we demonstrate the application of probe capture enrichment sequencing for molecular fine-typing *N.m*. directly from biologically complex oropharyngeal carriage specimens, improving the data capture of future *N.m*. surveillance studies.

## Data Summary

Raw sequence data for all samples presented in this study are available via the National Center for Biotechnology Information (NCBI) sequence read archive (BioProject: PRJNA1237918, see Table S1 for accessions). Source code is available at https://github.com/RColdbeck-Shackley/direct_WGS-of-Nmeningitidis.

## Introduction

*Neisseria meningitidis* (*N.m*.) is the causative agent of invasive meningococcal disease (IMD), a potentially fatal disseminated infection that causes meningitis and septicaemia. Although the incidence of IMD in Australia is rare (0.47 per 100,000 cases in 2015), it is associated with high mortality rates in children and infants, with a second peak occurring during adolescence corresponding to increased carriage rates in these demographics [1,2].

Asymptomatic carriage of *N.m*. in the oropharynx is common in adolescence and young adulthood [3]. Asymptomatic carriers of *N.m*. hypervirulent strains are the most significant reservoir for new IMD cases [3,4]; thus, monitoring carriage in the community is informative for public health interventions.

Certain *N.m*. strains are known risk factors associated with an individual developing IMD [5]. Meningococci have historically been categorized by serogroup based on antibody-mediated detection of capsule antigens. In Australia, the capsule type (genogroup) is primarily determined by PCR detection of serogroup-specific capsule biosynthesis genes. However, PCR-based methods have limited resolution, cannot identify commonly carried non-groupable strains and require multiple assays to determine sub-capsular strain variation [6,7]. Sub-capsular typing (fine typing) enables identification of hypervirulent strains more likely to cause disease, enhances epidemiological discrimination, improves outbreak traceability compared to genogroup alone, can identify likely capsule switching events and can predict vaccine coverage [6,8,10].

Whole-genome sequencing (WGS) has streamlined *N.m*. fine typing since it enables assessment of multiple typing schemes simultaneously. This includes characterization of the *porA* and *fetA* variable regions and *Neisseria* multi-locus sequence typing (MLST) based on seven core-genes under stabilizing selection (*abcZ*, *adk*, *aroE*, *fumC*, *gdh*, *pdhC* and *pgm*) [8]. MLST sequence types (ST) can be further clustered into clonal complexes (cc) that are useful for identifying common disease-causing strains [11]. Most IMD in Australia are associated with hyperinvasive strains, such as genogroup B (MenB) cc41/44, MenY cc23 and MenW cc11 [12,13]. Additionally, the *Bexsero* Antigen Sequence Typing (BAST) scheme classifies variants based on the fHbp, NadA, NHBA and PorA vaccine antigens and is used to predict vaccine efficacy of the MenB vaccines Bexsero and Trumenba [14].

Although WGS is useful for *N.m*. fine typing, it is normally limited to cultured isolates. *N.m*. is notoriously susceptible to temperature and other environmental stressors, often resulting in suboptimal culture rates [15,16]. Recently, we evaluated the effect of the 4CMenB vaccination on the genomic epidemiology of *N.m*. carriage from the ‘B Part of It*’* study, an Australian cluster randomized controlled trial conducted between 2017 and 2020 in South Australia (SA) [6], and then ‘B part of it NT’, an observational study in the Northern Territory (NT) was conducted in 2021 to 2024 [17]. Despite optimized sample storage and culture conditions [18,19], ~30% of samples that screened positive for *N.m*. by *porA* PCR from the SA ‘B Part of It’ study were unculturable. Sample viability was even lower for NT samples, likely due to extra transport distances and hot weather conditions in this part of Australia.

Methods for direct sequencing of pathogens from clinical specimens are now available, but challenges exist. Primary specimens often contain high concentrations of nucleic acid from the host and/or other microbes compared to the pathogen of interest. Deep sequencing and metagenomic analysis can improve sequencing depth of the pathogen of interest but are expensive and often ineffective at resolving subspecies variation from highly microbially diverse samples [20]. Alternative methods utilize PCR amplification or probe-capture enrichment of target pathogen nucleic acid from metagenomic sequencing libraries [21,22]. Probe capture enrichment WGS has been successfully applied to IMD samples from sterile sites (blood, cerebrospinal fluid (CSF)) for capsular transport gene (*ctr*A) PCR up to a cycle threshold (Ct) of 35 [22]. However, it has not been attempted on microbiologically complex samples like the oropharyngeal specimens used in carriage studies [23].

This study aimed to determine the sensitivity of a direct probe-capture enrichment WGS (dWGS) method compared to isolating WGS for fine-typing *N.m*. carriage strains from oropharyngeal swabs using currently available bioinformatic tools. Further, we use dWGS to characterize unculturable *N.m*. collected as part of the SA and NT ‘B part of it*’* carriage studies.

## Methods

### Sample selection

A total of 161 PCR confirmed oropharyngeal swab specimens with *porA* Ct≤35 were selected for testing from the ‘B Part of It’ (*n*=137) and ‘B part of it NT’ (*n*=24) studies conducted in the Australian jurisdictions of SA and the NT, respectively. Initially, specimens were processed and *Neisseria* culture was attempted as previously described [24]. Following initial culture attempts, specimens were transferred to deep well storage blocks and frozen at −80 ^o^C until their use in this study. From South Australian participants, 114 samples were unable to be cultured (unculturable) [6]. For the remaining 23 SA samples, isolates were previously cultured (culturable) and sequenced by WGS, with typing results available for comparison [25]. All 24 NT samples were unculturable.

### Nucleic acid extraction and *porA* PCR detection screening

External lysis buffer (Roche) was added to the swab liquid in a 1:5 ratio with a minimum of 200 µl final volume for extraction. Total DNA was extracted using the QIASymphony SP with QIASymphony DSP Virus/Pathogen Kit (QIAGEN, Hilden, Germany) according to the manufacturer’s instructions including the use of carrier RNA. DNA concentration was quantified using Quant-IT dsDNA High Sensitivity Kit (Thermo Fisher Scientific, MA, USA). Extracts were screened for the presence of *N.m*. by *porA* PCR using a previously published assay [26].

### NGS library preparation, probe-capture enrichment and sequencing

Library preparation and probe-capture enrichment were performed using the Agilent SureSelect XT Low Input Target Enrichment system following the recommended protocol including modifications for sequencing of viruses and bacteria. Specifically, *N.m* community design probes (5,191–6,712, Agilent) were diluted 1:10 prior to use, and 2 µl of diluted probe was added to individual sample hybridization reactions. Pre- and post-capture PCR were performed for 13 cycles and 18 cycles, respectively. PCR clean-up was performed using AxyPrep MAG PCR Clean-Up Kit (Corning). DNA was quantified using Quant-IT dsDNA High Sensitivity Kit (Thermo Fisher Scientific), and library size was determined using a DNA 1 K/12 K/Hi Sensitivity Assay LabChip (Perkin Elmer). Post-capture libraries were pooled using equimolar ratios in batches of 32 including a negative control to assess for contamination and sequenced on a NextSeq 550 platform with NextSeq 500/550 Mid-Output Kit v2.5 (300 cycles) (Illumina Inc.). Index masking was performed using i8 and i10 parameters and paired-end sequencing was performed for 2×150 cycles.

### Metagenomic next-generation sequencing bioinformatic processing

For metagenomic analyses, Agilent SureSelect XT pre-capture libraries were pooled in equimolar ratios and sequenced using the NextSeq 500/550 High Output Kit v2.5 (300 Cycles). Raw fastq paired-end reads were processed using an in-house metagenomic pipeline. Briefly, this pipeline performs read trimming and deduplication with *fastp* (v0.22.0) [27], host read removal by mapping to the human reference genome (GenBank: GCA_000001405.15 GRCh38) with *bowtie2* (v2.2.5) and filtering with *samtools* (v1.13). Microbiome profiling was determined using the *k*-mer taxonomic classifier *kraken2* (v2.1.2) [28,30].

### Direct WGS probe-enriched bioinformatic processing and typing

Paired-end raw fastq files were processed using *trimmomatic* (v0.39) to remove Illumina adapters and low-quality bases using a 4:20 sliding window and minimum length 30 [31]. Next, trimmed reads were aligned to the human reference genome (GenBank: GCA_000001405.15 GRCh38) using *bowtie2* (v2.2.5) and filtering with *samtools* (v1.13). Filtered reads were then built into contiguous assemblies using the *shovill* pipeline with *spades* (v1.1.0) and their quality assessed by *Seqtk* (v1.3-r106) packaged as *fa* from the *nullarbor* pipeline [32,33]. Assemblies were aligned to a *N.m*. reference (GenBank: GCA_008330805.1 ASM833080v1) using *lra* (v1.3.2) and filtered using *samtools* (flags -F 4 and -F 2048) to remove contaminant contigs [34]. Filtered assemblies were input to *mlst* (v2.19.0), *meningotype* (v0.8.5) and *characterise_neisseria_capule* tools for in-house typing [35,37]. Filtered assemblies for samples that passed quality control (QC) assessment were also submitted to and are available via the pubMLST isolate database to confirm typing results. Genogroup was determined using the consensus of *meningotype*, *characterise_neisseria_capule* and pubMLST database results.

### Sample species identification and QC assessment

Sequencing quality was assessed using *Seqtk* (v1.3-r106) packaged as *fq* from the *nullarbor* pipeline on host-filtered reads for sequencing read data statistics and *kraken2* (v2.1.2; database: k2_pluspf downloaded on 20220607) for species identification [33]. Briefly, samples were considered as failed QC if the percentage of reads mapping to *N.m* was not the most abundant species identified or if there were fewer than 500,000 total *N.m* reads. Samples were also considered as failed QC if the second most abundant species was another *Neisseria* and the percentage of *N.m* reads was less than 40%.

### Sensitivity calculations

WGS was performed on *N.m* isolates cultured from 23 specimens, according to Leong *et al.* [6]. Isolate sequences were then compared to their respective dWGS sequences that passed QC assessment, specifically looking at the genogroup, MLST, porA typing, fetA and BAST typing results. True positives (*TP*) were defined as concordant scheme types identified by both assays. False negatives (*FN*) were recorded if the scheme type was identified by WGS but was unidentified by dWGS (i.e. no exact match was identified in the pubMLST database due to missing or multiple loci being detected). Sensitivity (*S*) was calculated using the formula: S=TP÷(TP+FN). No false positives or true negatives were recorded precluding specificity calculations.

### Phylotyping

Samples that passed QC assessment were also further used for a phylotyping approach using core SNP alignments generated using *snippy* (v4.6.0) with filtered reads mapping to a reference (RefSeq: GCA_008330805.1) on default settings [38]. One hundred and twenty-two high-quality *N.m* reference sequences were downloaded from the NCBI RefSeq database and were used as contextual samples and processed using *snippy* with contig settings (see Table S2 for accession numbers, available in the online Supplementary Material). A full core SNP alignment including all samples and references was then generated with *snippy-core* (v4.6.0) and used as input for *FastTree* (v2.1.11) with a gtr+gamma model [39]. The resultant tree was visualized and annotated using *ggtree* (v3.6.2) [40].

### Statistical analyses

Data distributions were assessed using the Shapiro–Wilk test. For comparisons between multiple groups of equal size, Welch’s ANOVA with Games–Howell multiple comparisons was used. For comparison between two groups of unequal size, the Wilcoxon rank sum test was used. All statistical analyses were performed in *R* (v4.2.2).

## Results

### Enrichment of *N.m*. sequences from oropharyngeal samples by dWGS

**Fig. 1.**
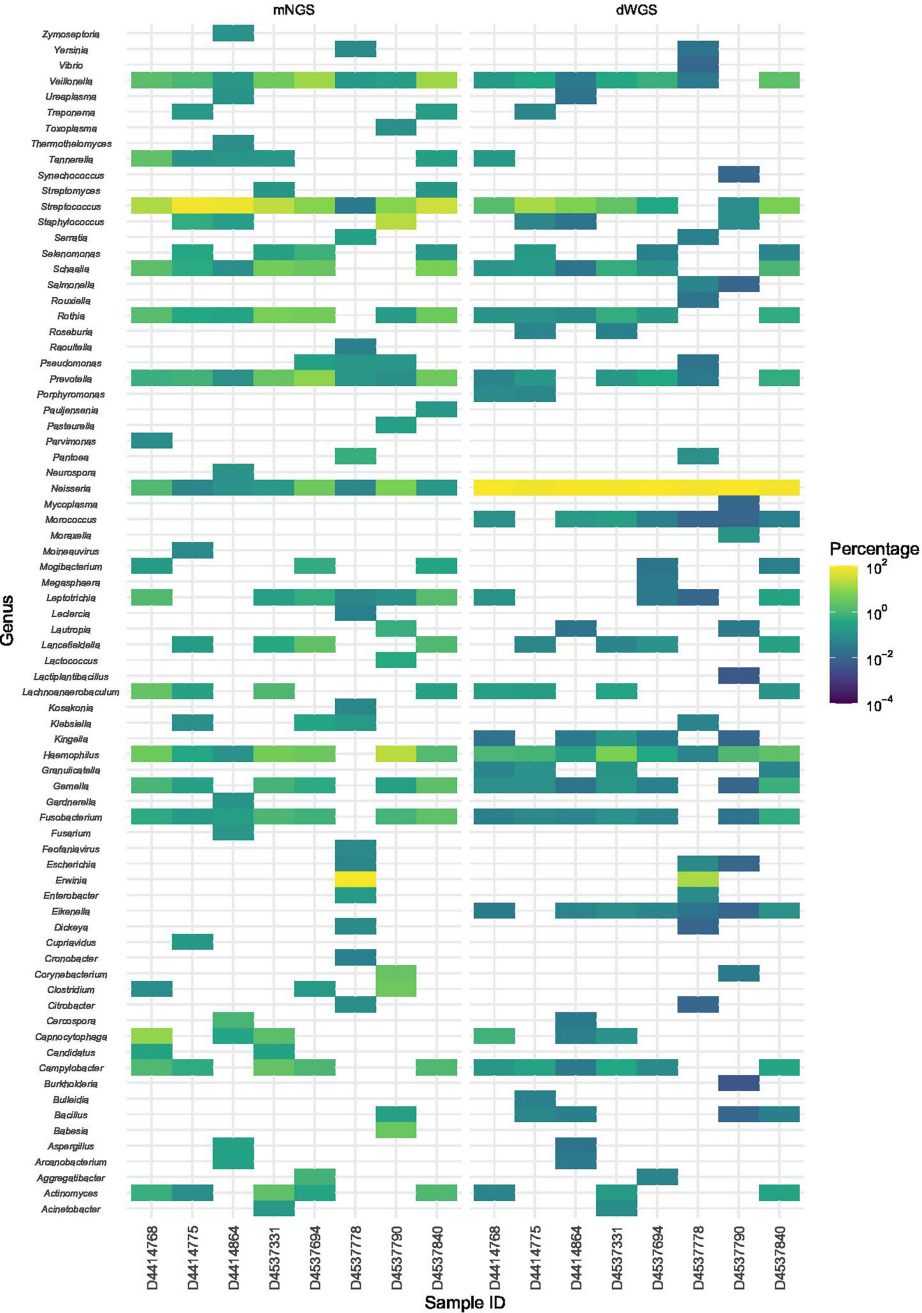
The 20 highest represented genera in pre-capture metagenomic (mNGS) or post-capture enriched (dWGS) sequencing libraries from oropharyngeal samples with detectable *N.m*.

To determine if dWGS enriched for *N.m.*, species identification was performed on pre-capture metagenomic [metagenomic next-generation sequencing (mNGS)] and post-capture (dWGS) sequences from the same library preparation in parallel for a subset of the unculturable samples (*n*=8). Fig. 1 shows that the pre-capture libraries contained sequences from diverse commensal bacteria. *Neisseria* was represented in the top 20 genera by percentage in pre-capture libraries for all eight samples but was present in lower proportions compared to other genera such as *Streptococcus*, *Haemophilus* or *Rothia*. Following probe-capture enrichment, the percentage of *Neisseria* genus reads increased compared to other commensals in all post-capture samples.

**Fig. 2.**
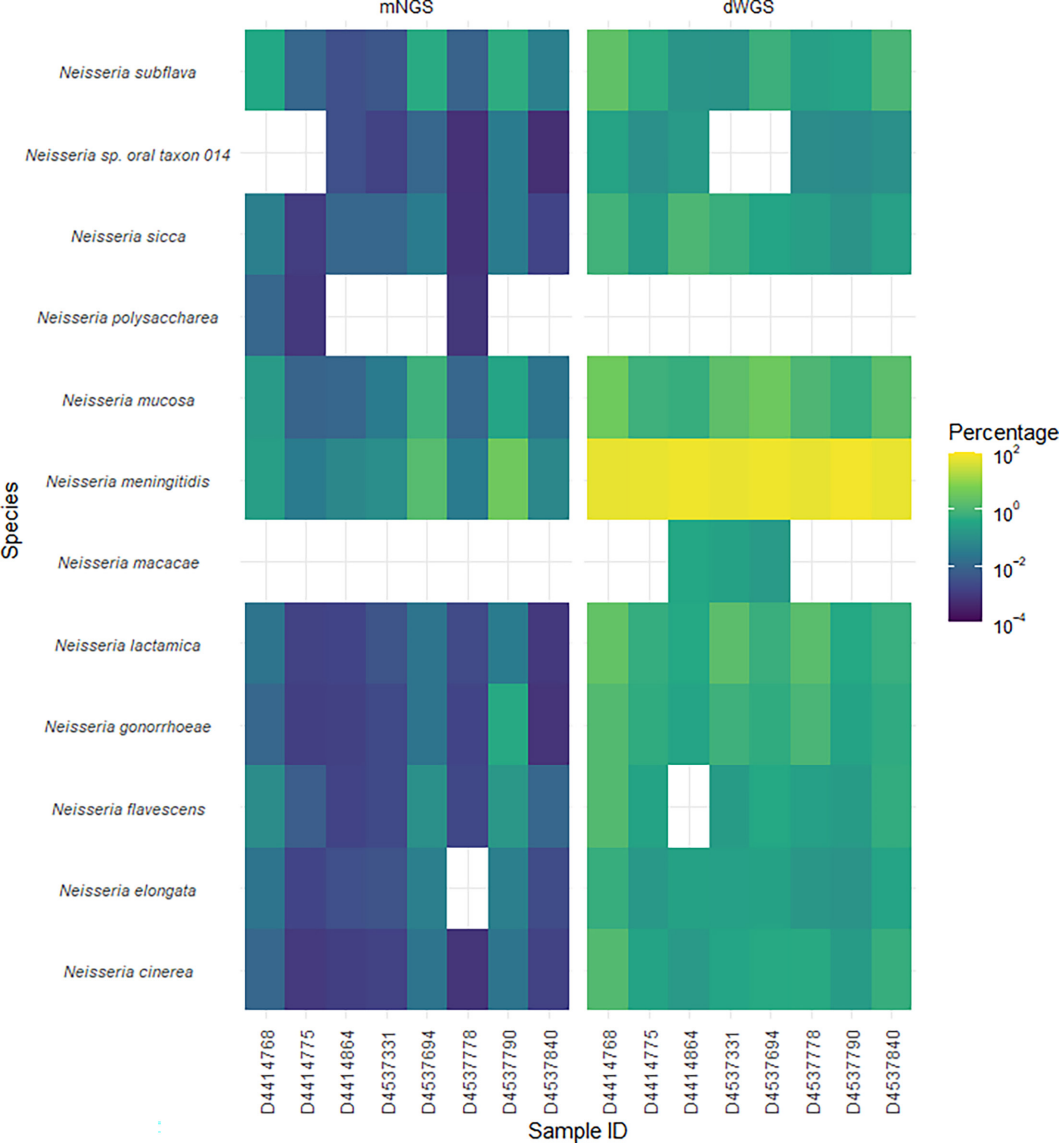
The percentage of reads mapping to the top ten most prevalent *Neisseria* species present in pre-capture metagenomic (mNGS) and post-capture enriched (dWGS) sequencing libraries.

**Fig. 3.**
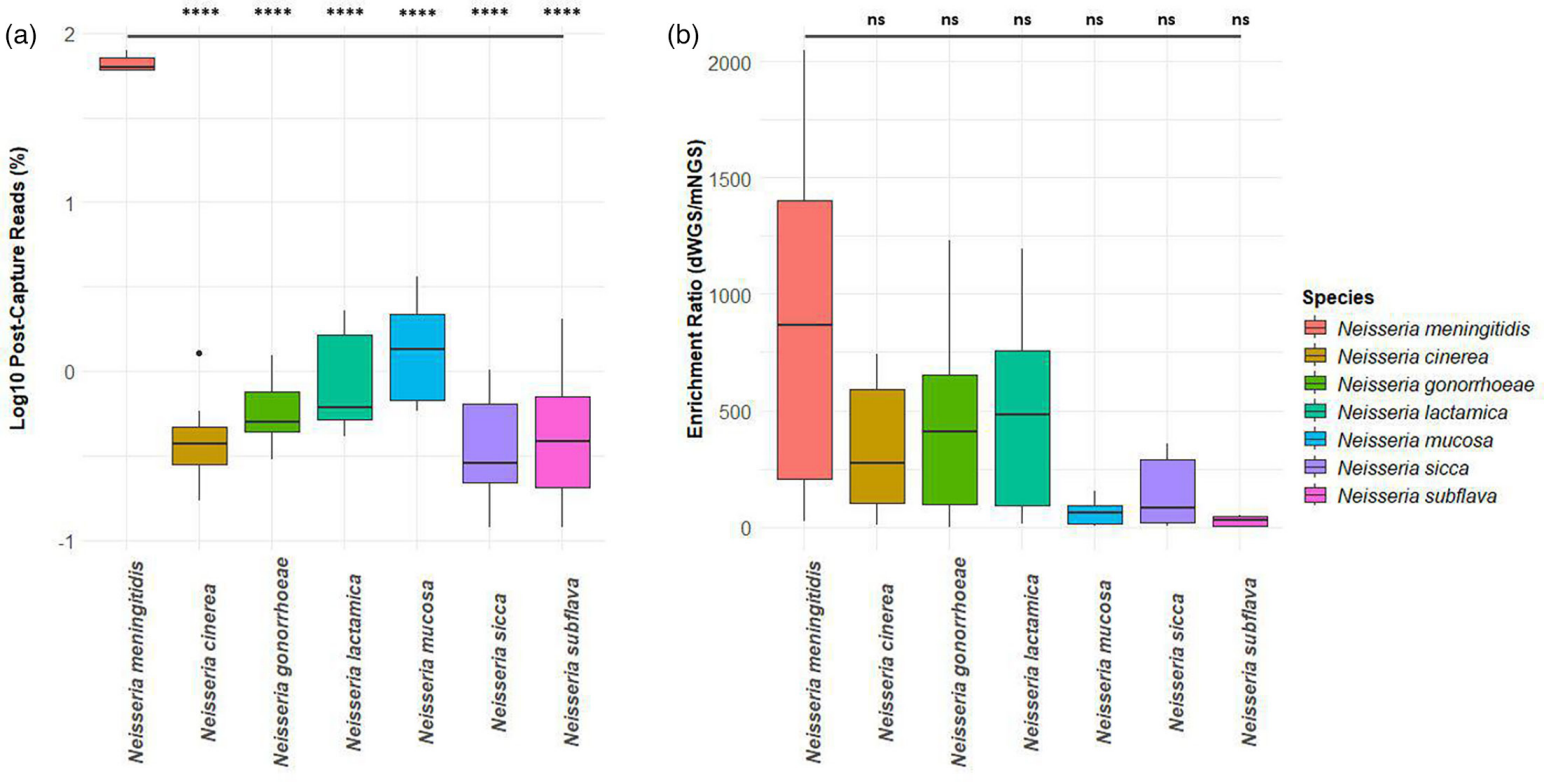
Pre- and post-capture reads were compared for the *Neisseria* species that were identified in all samples (*n*=8) sequenced in parallel by mNGS and dWGS, respectively. (a) Log10 transformed read percentages mapping to *Neisseria* species in post-capture libraries. (b) Read percentage enrichment ratios for *Neisseria* species were calculated by the read percentages from post-capture libraries (dWGS) divided by read percentages from pre-capture libraries (mNGS) independently for each species in each sample. Statistics were performed by Welch’s ANOVA with Games–Howell multiple comparisons. Statistical significance is summarized as follows for *P*-value: > 0.05 [non-significant (ns)], ≤ 0.05 (*), ≤ 0.01 (**), ≤ 0.001 (***), ≤ 0.0001 (****).

Next, the specificity of the enrichment process for *N.m*. compared to other *Neisseria* species was assessed. Fig. 2 demonstrates dWGS enriched for *N.m*. and, to a lesser extent, other *Neisseria* species. Although the percentage of post-capture *N.m*. reads was significantly greater compared to the other *Neisseria* (Fig. 3a), enrichment ratios were not significantly different between *Neisseria* species (Fig. 3b). Collectively, this demonstrates that the dWGS method enriched for *N.m*. sequences, but non-specific enrichment of other *Neisseria* species also occurred.

### Quality control assessment of probe-capture enriched dWGS samples

**Fig. 4.**
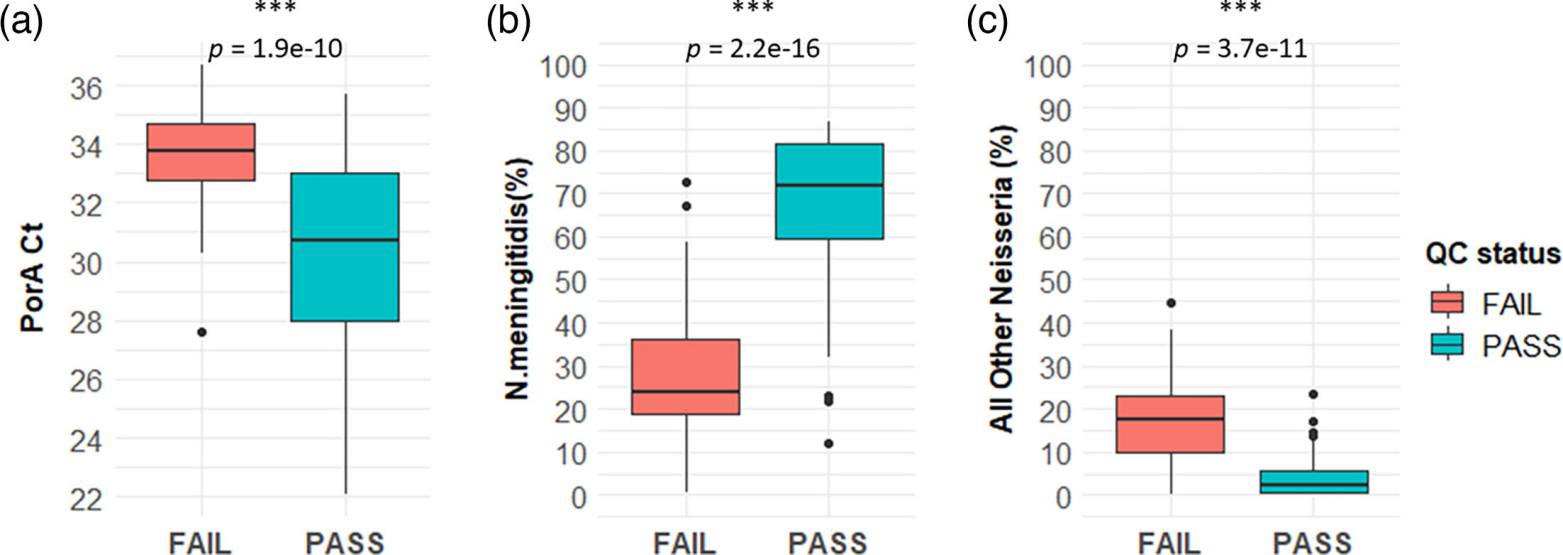
Factors associated with samples that failed or passed quality control assessment. Samples failed QC if they met the following criteria: the top species identified ≠ ‘*Neisseria meningitidis*’, or the number of *N.m* reads≤500,000, or the second species identified=other *Neisseria* species and the proportion of *N.m* reads≤40 %. (**a**) The *porA* PCR Ct of DNA extract, (**b**) the percentage of *N.m*. reads in enriched dWGS libraries identified and (c) the percentage of other *Neisseria* species reads in enriched dWGS libraries. Statistical comparisons were performed by the Wilcoxon rank sum test.

**Fig. 5.**
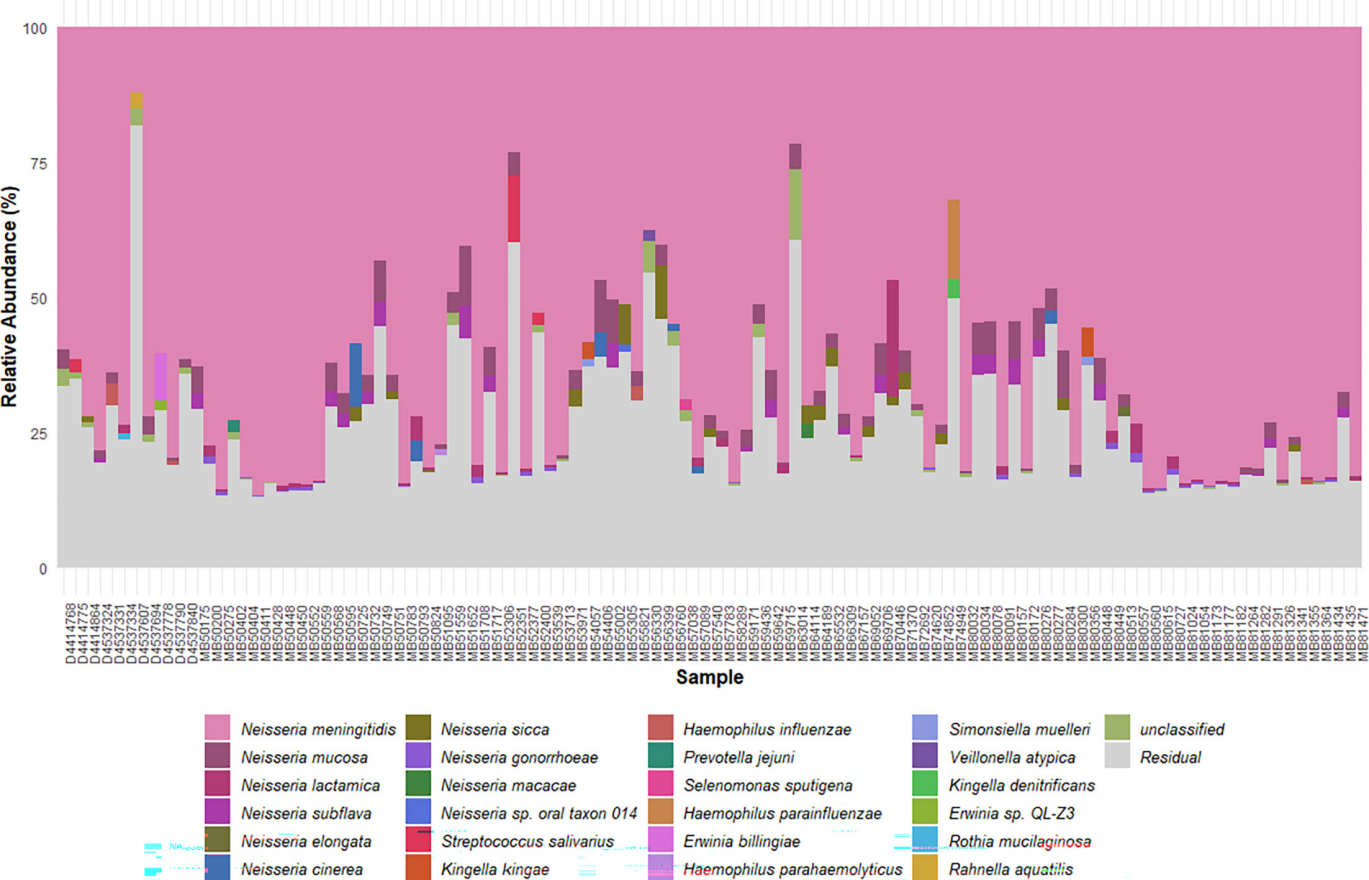
Top three species identified in post-capture enriched dWGS sample libraries that passed QC assessment. Samples failed QC if they met the following criteria: the top species identified ≠ ‘*Neisseria meningitidis*’, or the number of *N.m* reads≤500,000, or the second species identified=other *Neisseria* species and the proportion of *N.m* reads≤40 %. Species identified in the top three are coloured, and ‘Residual’ (grey) represents all other low-prevalence species identified in these samples.

Since the dWGS approach also enriched other *Neisseria* species, we implemented a quality control (QC) assessment that excluded samples with a high proportion of non-meningitidis reads from further analysis. The factors associated with samples that passed or failed this QC assessment were then analysed. Following dWGS, 107 out of all 161 probe-capture enriched samples passed QC assessment (66.46%; 95% CI: 58.54–73.58%). The remaining 54 samples that failed QC assessment were excluded from further analyses (see Table S1 for all QC data). Samples that passed QC had a significantly lower median *porA* Ct compared to those that failed (median: PASS=30.69, FAIL=33.78; Wilcoxon *P-*value=1.871e−10) (see Fig. 4a). Likewise, the percentage of *N.m*. reads was higher in samples that passed compared to those that failed QC assessment (median: PASS=72.02%, FAIL=24.07%; Wilcoxon *P*-value=2.2e−16) (see Fig. 4b). Other *Neisseria* species were frequently identified amongst the top three species in samples irrespective of their QC status (frequency: PASS=(97/107) 90.65%, FAIL=(46/54) 85.19%, OR=1.68; 95% CI: 0.54–5.09, Fisher’s *P*-value=0.3022). Despite their frequent identification, the percentage of other *Neisseria* species reads was lower in samples that passed QC assessment compared to those that failed (median: PASS=2.24%, FAIL=17.62%; Wilcoxon *P*-value=3.689e−11) (Fig. 4c). Enriched samples passing QC assessment frequently had non-*Neisseria* detected in the top three species including common commensal *Haemophilus*, *Kingella*, *Prevotella* and *Streptococcus* species [23] (see Fig. 5). No association was found between the presence of any non-*Neisseria* species in the top three with a sample passing QC assessment.

### Specificity of direct WGS compared to isolated WGS typing results

**Fig. 6.**
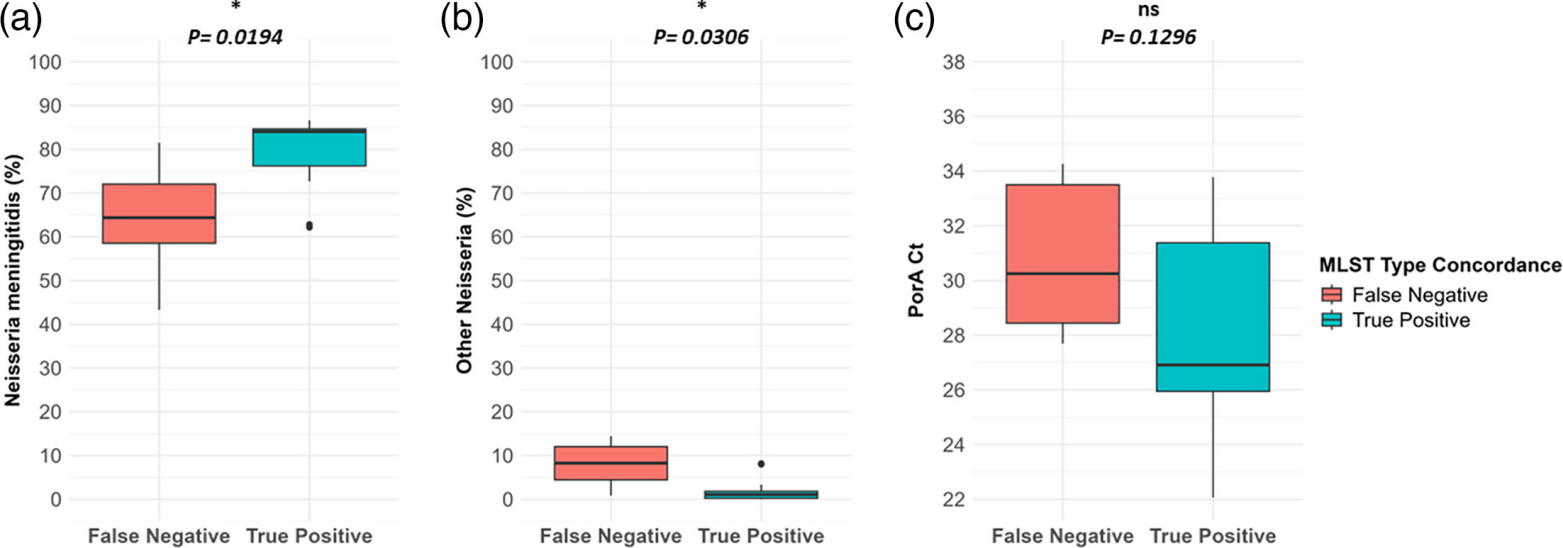
Factors associated with correct identification of MLST type for dWGS compared to isolate WGS for all carriage specimens with a corresponding isolate (*n*=17). (**a**) The percentage of reads in enriched dWGS libraries matching *Neisseria meningitidis*, (**b**) the percentage of reads in enriched dWGS libraries matching other *Neisseria* species and (c) *porA* PCR Ct of DNA extract. Statistics were performed by the Wilcoxon rank sum test.

After establishing a QC assessment that reduced read contamination from other *Neisseria* species, the sensitivity of dWGS molecular typing was analysed compared to isolate WGS. Of the 23 culturable samples sequenced previously by isolate WGS [6], 17 passed dWGS QC. Compared to respective isolate WGS typing, the genogroup identified by dWGS was true positives for 15 samples (S=88.23%; 95% CI: 62.25–97.93%), MLST for 12 (S=70.59%; 95% CI: 44.05–88.62%), *porA* for 17 (S=100%; 95% CI: 77.08–100.00 %), *fetA* for 16 (S=94.12%; 95% CI: 69.24–99.69 %) and BAST for 15 (S=88.23%; 95% CI: 62.25–97.93%) (see Table S3 for dWGS and WGS typing matrix). Of the 5 samples that had false negative results for MLST, 23 of 35 identified alleles were concordant with the isolate-derived MLST (S=65.71%) (see Table S4 for allele matrix). Sensitivity for MLST was significantly associated with samples having a higher percentage of *N.m*. (Fig. 6a) and a lower percentage of other *Neisseria* species identified (Fig. 6b). There was no significant association with *porA* Ct and MLST sensitivity results (Fig. 6c). There were insufficient false negative results to statistically analyse sample factors contributing to sensitivity for the other typing schemes. Collectively, this demonstrates that dWGS can perform typing that is highly concordant with isolate WGS results. However, a lower percentage of *N.m*. and higher proportions of other *Neisseria* species in post-capture libraries reduced MLST typing sensitivity.

### Typing of unculturable samples by dWGS

After assessing the sensitivity of dWGS typing, this approach was applied to all remaining unculturable samples that passed QC assessment (*n*=89). *PorA* and *fetA* were characterized for 85 and 76 unculturable samples, respectively (*porA*, 95.50%; *fetA*, 85.39%). Thirty-three different *porA* types were identified (for a full summary of typing results, see Table S5). The most common *porA* types were 5–1, 10–1 (*n*=10, 11.23%), followed by 22, 14 (*n*=8, 8.99%) and 22–1, 14 (*n*=7, 7.87%). *FetA* was less diverse with 20 types being identified, and the most common were as follows: F4-1 (*n*=14, 15.73%), F3-9 (*n*=13, 14.61%) and F5-5 (*n*=11, 12.34%).

Eighty-four samples had genogroup identified (94.38%). Most were non-groupable (*n*=33, 37.08%), followed by MenY (*n*=21, 23.60%), MenB (*n*=19, 21.85%) and cnl (*n*=7, 7.87%). Four MenC (4.49%) were also detected in these samples.

MLST was identified for 49 samples (55.06%), including 23 different STs from 11 cc. ST-35 cc35 (*n*=11, 12.36%), ST-1655 cc23 (*n*=5, 5.62%), ST-23 cc23 and ST-823 cc198 (*n*=4, 4.49%) were the most abundant types identified. Additionally, this analysis identified two specimens from cc41/44, which are linked to IMD cases in Australia [13].

BAST was identified for 71 samples (79.78%) with a total of 39 types identified in the dataset. The most common BASTs were 221 and 257 (*n*=7, 7.87%), 228, 261, 315 and 4307 (*n*=4, 4.49%). These had different MenDeVAR Bexsero Reactivity scores, and BAST 315 had an exact match to the vaccine antigens, indicating MenB vaccination may provide protection against invasive disease caused by this variant. Two hundred sixty-one has no vaccine reactivity, and there were insufficient data for 221, 228, 257 and 4307 to determine reactivity based on experimental studies.

Although these numbers were small in comparison, the pattern of typing results was similar to previously analysed culture isolate WGS data from this study cohort [6].

**Fig. 7.**
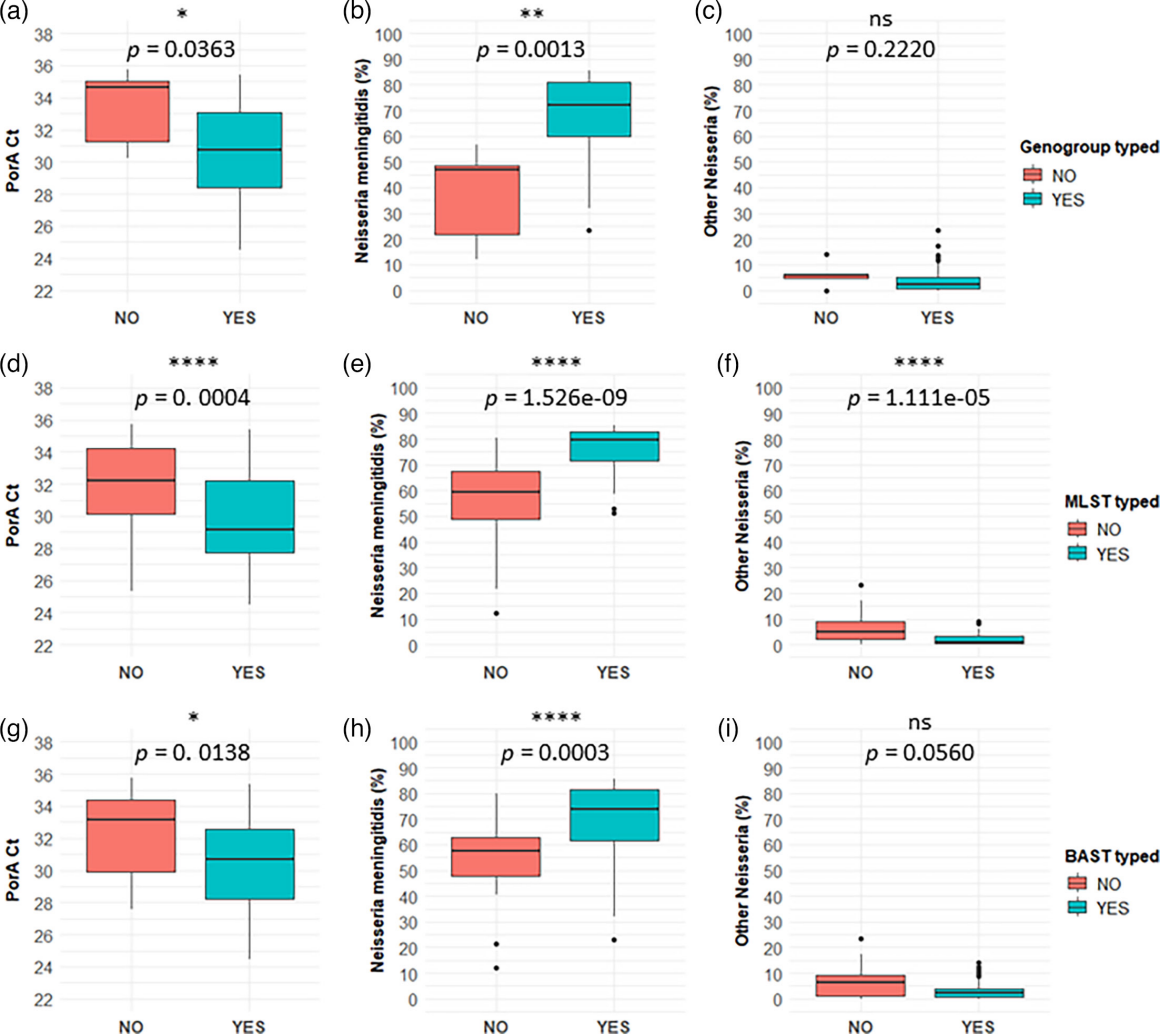
Factors associated with typeability of dWGS unculturable carriage specimens (*n*=89). (**a–c**) Genogroup was compared to *porA* Ct, the percentage of *N.m*. reads or the percentage of other *Neisseria* species in the sample. This comparison was similarly performed for MLST (**d–f**) and BAST (**g–i**) typing results. Statistics were performed by the Wilcoxon rank sum test.

For samples where typing results were unidentified (i.e. no exact database match due to a partial match, missing or multiple allele assignments), we postulated contributing factors were the amount and proportion of *N.m*. in the sample, and the presence of closely related *Neisseria* species, as this may interfere with typing due to sequence similarity. To interrogate this assertion *porA* Ct, the percentage of *N.m*. in sample sequence reads and the percentage of all other *Neisseria* species, read contaminants in ‘typed’ (YES) and ‘untyped’ (NO) samples were compared for genogroup, MLST and BAST typing schemes (see Fig. 7a–i).

Genogroup and BAST were associated with the amount (*porA* Ct) and proportion of *N.m*. [*N. meningitidis* (%)] but were not significantly associated with the proportion of other *Neisseria* species [other *Neisseria* (%)] in these samples. However, for MLST, there was a significant correlation for all three factors.

Identification rates for each locus within the BAST and MLST schemes were also assessed using the pubMLST isolate search and annotation quality metrics score for all untyped samples (BAST, *n*=18; MLST, *n*=40). This analysis revealed suboptimal identification for all loci in the MLST and BAST typing schemes, especially for *fumC* and NHBA loci where only 35% and 56% of the untyped samples, respectively, had a known allele identified (see Table S6 for all loci).

**Fig. 8.**
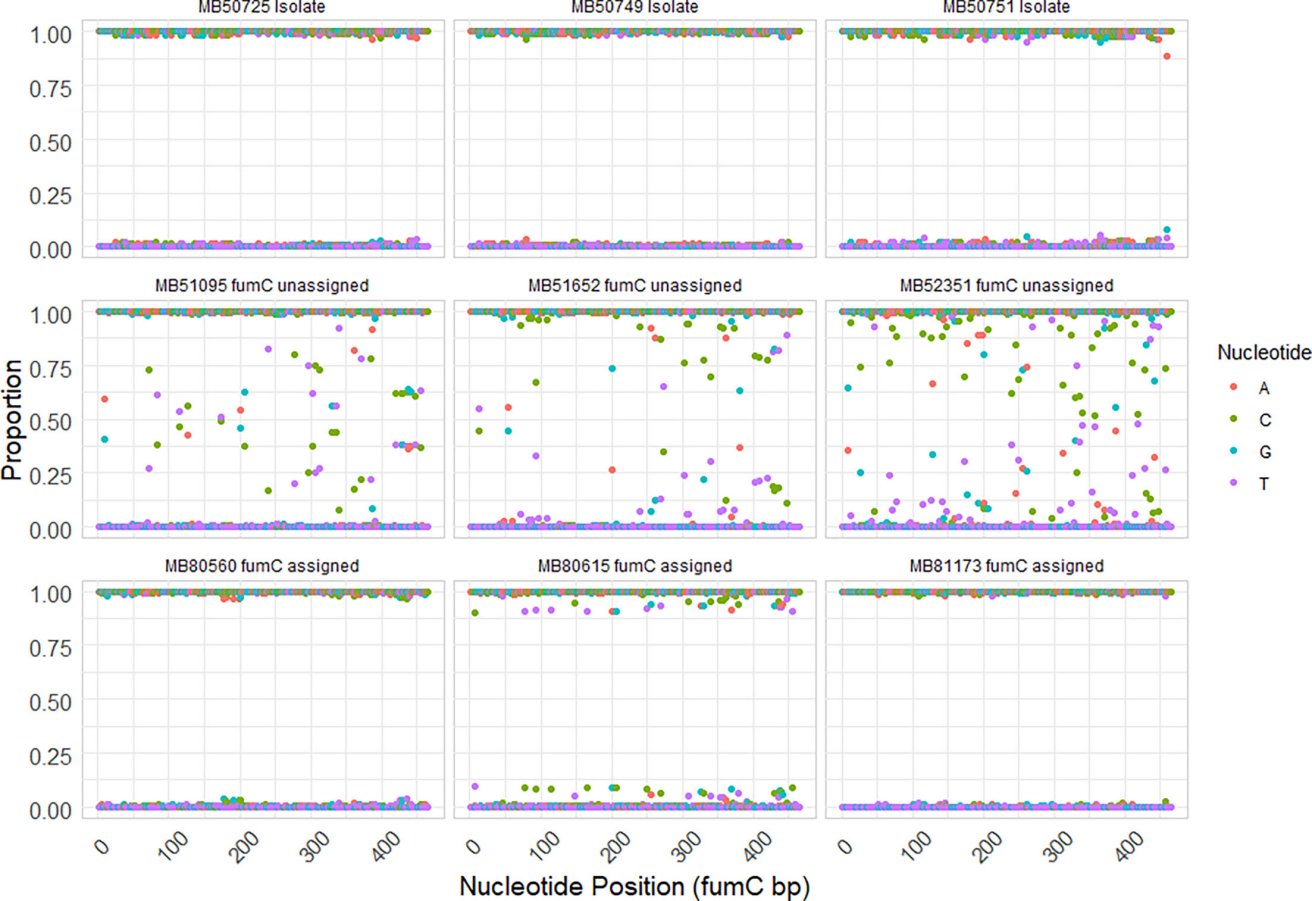
Nucleotide proportions of filtered reads aligned to the *fumC* loci from representative isolates sequenced by WGS (isolate), samples sequenced by dWGS that did not have an exact *fumC* allele match in the pubMLST database (*fumC* unassigned) or samples sequenced by dWGS with an exact *fumC* allele match (*fumC* assigned).

The *fumC* locus was further investigated as it had the lowest identification rate. Filtered reads from three representative isolates sequenced by WGS, or three dWGS samples with or without the *fumC* allele assigned, were aligned to their respective closest *fumC* allelic sequences. Base frequencies were then assessed at each nucleotide position in the *fumC* locus. Fig. 8 shows that for dWGS samples with unassigned *fumC* alleles, the *fumC* locus exhibited heterogeneous base proportions. In contrast, the *fumC* locus from samples with assigned alleles was more similar to isolate WGS sequences, displaying relatively homogeneous nucleotide proportions, with the majority of bases being more than 80% composed of a specific nucleotide. In addition, visual inspection of read alignment samples without *fumC* assignment revealed variant bases were grouped on separate reads, suggesting that there were multiple *fumC* allele sources in the original sample.

### Phylotyping as an alternative method for cc estimation

**Fig. 9.**
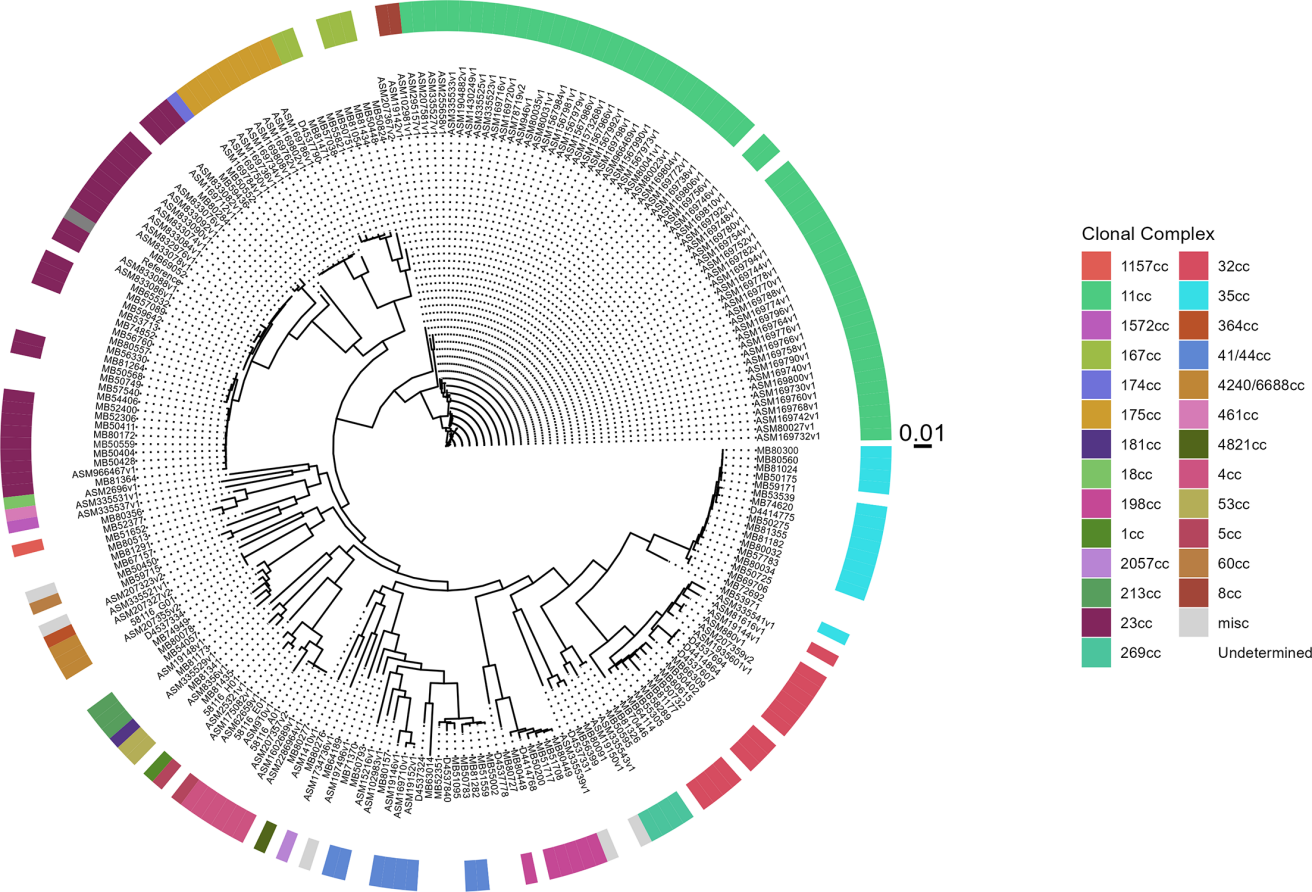
A full core SNP alignment, including all samples passing QC and representative sequences from diverse cc, was used as input for phylotyping. A tree structure was built using *FastTree2* -gtr -gamma settings. Samples are coloured according to their cc derived from their previously assigned MLST type. ‘Undetermined’ samples (white) had no identified MLST or cc due to having been previously assigned incomplete or partial MLST allelic profiles. The tree scale indicates evolutionary distance.

Since some samples remained without an MLST assigned due to having an incomplete MLST allelic profile characterized by fine typing, another approach was investigated. Classification at the cc level was attempted by phylogenetic analysis (phylotyping) for all samples that passed dWGS QC assessment (*n*=107). Fig. 9 shows that samples formed distinct clades separated by cc. All samples that had MLST assigned by fine typing were positioned on branches consistent with reference sequences in the correct cc. Samples without MLST assigned (*n*=44) also clustered amongst these MLST-assigned samples and references. For all samples without MLST assigned, an estimated cc (phylotype) was inferred based on the cc of other members of the clade. The phylotype was then compared against each respective partial MLST allelic profile (see Table S7 for allele profile comparison). Phylotypes were concordant with respective partial MLST profiles in 37 of 44 samples without MLST assigned by fine typing (84.01%). However, seven of these samples remained unresolved by phylotyping. Of these samples, two were branched as a single sample within a clade, precluding phylotype assignment, and five had MLST allelic profiles discordant with phylotype results. Nevertheless, phylotyping correctly assigned cc in 100 of the 107 dWGS samples (S=93.46%, 95% CI: 86.51–97.10%).

## Discussion

*N.m.* is notoriously sensitive to environmental and storage conditions leading to high rates of culture failure and hampering isolate WGS in carriage studies [16]. Previous studies have demonstrated ~70% sensitivity for *N.m*. culture from oropharyngeal or throat swabs, leaving ~30% of samples that cannot be cultured [18,25]. Here, we describe a method to sequence and perform fine typing of *N.m*. directly from unculturable oropharyngeal carriage specimens.

Unlike blood and CSF samples from IMD cases where *N.m*. is the only organism present in these normally sterile sites [22], our study demonstrated oropharyngeal specimens are microbiologically complex, including the presence of nucleic acids from other *Neisseria* species even after probe-capture enrichment. Comparison between pre- and post-capture sequencing revealed that although nucleic acid from most commensal bacterial genera was depleted by dWGS, *N.m*. sequencing reads could not be further enriched from closely related *Neisseria* species. This is indicative of probe cross-reactivity and supports the close genetic relationships between *Neisseria* species that are often poorly differentiated by traditional phenotypic and genetic analyses [41]. Future studies that optimize probe-library composition, hybridization conditions or that develop bioinformatic tools better able to differentiate between related *Neisseria* species would greatly benefit this area of research.

This study demonstrated that the main factor impacting typing by dWGS was the proportion of *N.m*. sequencing reads in post-capture libraries. Unfortunately, this can only be determined post-sequencing and cannot be used as a sample selection criterion. However, based on the interquartile range of typed samples in this study, it is likely that a more stringent QC cut-off for *N.m*. read percentage, such as ~60–80%, would increase typing sensitivity. Although *porA* Ct was a strong indicator of whether samples passed or failed QC, it was only moderately associated with typeability for multi-locus schemes using existing bioinformatic typing tools, especially for genogroup or BAST. Unlike for CSF and blood specimens sequenced using a similar approach [22], this lack of a strong association between sequencing outcome and pre-screening PCR Ct likely reflects the microbiological complexity of oropharyngeal specimens. Although pre-screening based on *porA* Ct excludes some data loss, it is not the sole predictor of sequencing outcome in complex specimens. However, based on these results, a lower cut-off Ct for oropharyngeal specimens should be introduced to maximize sequencing efficiency. In this study, the median Ct for samples that passed QC assessment was ~Ct 30; this could be used as a guide for sample selection in future dWGS carriage studies.

MLST results were also significantly impacted by the presence of other *Neisseria* species, reflecting that the MLST scheme targets highly conserved loci across all *Neisseria* species [8]. Indeed, analysis of the *fumC* locus showed that these oropharyngeal samples are more likely to have multiple *fumC* alleles, likely sourced from other *Neisseria* species. Significant reductions in NHBA allele identification were also observed despite BAST not being significantly associated with the presence of other *Neisseria* species (*P*=0.05599). This may be due to the relatively small sample size in this study. Notably, a study by Muzzi *et al*. demonstrated that fHbp and NHBA antigens are conserved amongst many *Neisseria* species, whereas NadA was only found in *N. cinerea*, potentially explaining differences in locus identification [42]. The same study also demonstrated intra-genus variability of NHBA was akin to the level of variability amongst *N. meningitidis*. Together with results from our study, it suggests that BAST is likely impacted by the presence of other *Neisseria*, but possibly to a lesser extent compared to the highly conserved MLST scheme. Despite this, the ability of dWGS to determine BAST adds important information regarding the predicted MenB vaccine reactivity in unculturable carriage samples not otherwise able to be captured by isolate WGS. Unlike the MLST and BAST typing alleles, capsule expression was historically considered a unique feature of *N. meningitidis*, as this is the key virulence determinant for this pathogenic *Neisseria* species [5]. However, recent studies have identified homologues of capsule expression genes in multiple non-pathogenic *Neisseria* species [43]. Despite this, we found no evidence that these homologues impacted genogroup results that could be identified in ~94% of unculturable samples. Sequence similarity may also affect direct sequencing of other *Neisseria* species, such as *Neisseria gonorrhoeae*, in microbially complex specimens like the urogenital tract, where other *Neisseria* species can also colonize or cause infections [44,45]. Future studies should be interpreted accordingly. Aside from multi-locus typing schemes, the assessment of the *porA* variable region produced the most consistent typing results, likely owing to it being uniquely expressed by *N.m*. despite pseudogenes existing in other *Neisseria* [46]. This may be a useful typing scheme for dWGS carriage studies in the future.

Despite the influence of other *Neisseria* contaminant reads on *N.m*. fine typing results, we observed a strong phylogenetic signal that categorized samples at the level of the clonal complex. This phylotyping approach may prove to be a useful secondary analysis where fine-typing results are not resolved by other approaches. This is likely due to the filtering of sequences from other *Neisseria* species during the time of read alignment to the *N.m*. reference genome and the consideration of additional genomic regions aside from highly conserved typing loci.

This study has limitations. Aside from the small sample size that limited the statistical significance of some analyses, since multiple *N.m*. strains can co-habit the oropharyngeal and nasopharyngeal sites [3,16], it could not be ruled out that this also impacted typing results. Despite capturing a wide variety of strains, not all genogroups were represented in the data, as many commensal *N.m*. strains are unencapsulated; therefore, we do not know if this method is biassed towards typing of more commonly sequenced genogroups.

Collectively, our data demonstrate that dWGS is a viable method to analyse unculturable *N.m*. oropharyngeal specimens. Since the meningococcal types identified in these unculturable specimens were consistent with published results and fewer in number than those from previously sequenced cultured isolates, this study does not alter any conclusions from the ‘B part of it’ genomic analysis study [25]. However, this approach enabled the characterization of meningococci to a level equivalent to clonal complex for ~93.46% of unculturable samples that were previously unable to be sequenced using standard WGS. This adds to work by others using similar methods, enabling the sequencing of uncultured IMD clinical specimens [22]. However, due to the presence of other *Neisseria* species commonly present in oropharyngeal sites, this method is most suited to *N.m*. specific typing schemes such as genogroup or the *porA* gene and, additionally, for whole-genome phylogenetic approaches to typing.

## Supplementary material

10.1099/mgen.0.001464 Uncited Supplementary Material 1.

## Abbreviations

BAST: *Bexsero* Antigen Sequence Typing
cc: clonal complexes
CSF: cerebrospinal fluid
Ct: cycle threshold
dWGS: direct probe-capture enrichment WGS
IMD: invasive meningococcal disease
MLST: multi-locus sequence typing
mNGS: metagenomic next-generation sequencing
NCBI: National Center for Biotechnology Information
*N.m.*: *Neisseria meningitidis*
NT: Northern Territory
QC: quality control
SA: South Australia
WGS: whole-genome sequencing
